# A retrospective study for long-term oncologic and obstetric outcomes in cervical intraepithelial neoplasia treated with loop electrosurgical excision procedure: focus on surgical margin and human papillomavirus

**DOI:** 10.1186/s12905-024-02923-5

**Published:** 2024-02-12

**Authors:** Angela Cho, Min-Young Kim, In-Sun Park, Chul-Min Park

**Affiliations:** 1 Department of Obstetrics and Gynecology, Jeju National University Hospital, Jeju-si, Republic of Korea; 2 Department of Obstetrics and Gynecology, College of Medicine, Jeju National University, Aran 13-gil, Jeju-si, Jeju-do 63241 Republic of Korea; 3 Seoul National University Hospital, Seoul, Republic of Korea

**Keywords:** Loop electrosurgical excision procedure, Cervical intraepithelial neoplasia, Human papilloma virus, Margin status, Obstetric outcomes, Oncologic outcomes

## Abstract

**Background:**

The present study aimed to evaluate the long-term oncological and obstetric outcomes following the loop electrosurgical excision procedure (LEEP) in patients with cervical intraepithelial neoplasia (CIN) and investigate the risk factors for recurrence and preterm birth.

**Methods:**

This retrospective cohort study included patients who underwent LEEP for CIN 2–3 between 2011 and 2019. Demographic information, histopathological findings, postoperative cytology, and human papillomavirus (HPV) status were collected and analyzed. The Cox proportional hazards model and Kaplan-Meier curves with the log-rank test were used for risk factor analysis.

**Results:**

A total of 385 patients treated with the LEEP were analyzed. Treatment failure, including recurrence or residual disease following surgery, was observed in 13.5% of the patients. Positive surgical margins and postoperative HPV detection were independent risk factors for CIN1 + recurrence or residual disease (HR 1.948 [95%CI 1.020–3.720], *p* = 0.043, and HR 6.848 [95%CI 3.652–12.840], *p*-value < 0.001, respectively). Thirty-one patients subsequently delivered after LEEP, and the duration between LEEP and delivery was significantly associated with preterm-related complications, such as a short cervix, preterm labor, and preterm premature rupture of the membrane (*p* = 0.009). However, only a history of preterm birth was associated with preterm delivery.

**Conclusions:**

Positive HPV status after LEEP and margin status were identified as independent risk factors for treatment failure in patients with CIN who underwent LEEP. However, combining these two factors did not improve the prediction accuracy for recurrence.

## Background

Cervical intraepithelial neoplasia (CIN) serves as a precursor lesion of cervical cancer [1]. Patients with CIN 2–3 undergo the loop electrosurgical excision procedure (LEEP) to prevent progression [2]. However, previous studies have shown that women treated for high-grade CIN may still have an increased risk of recurrent CIN and cervical cancer for up to 25 years compared to the general population [2–5]. Therefore, screening for cervical cancer and Human papillomavirus (HPV) vaccination is crucial for these patients [6, 7].

HPV infection after LEEP is a well-recognized risk factor for disease recurrence [7]. Lesion size and severity, advanced age, and incomplete excision have been suggested as possible risk factors [1, 2]. However, according to previous studies, uncertainty remains regarding the factors or combinations of factors that most accurately predicts disease recurrence in patients with CIN following LEEP [2].

There is a scarcity of research specifically examining the long-term obstetric outcomes following LEEP. In addition, few studies have analyzed the risk factors for preterm birth such as depth of excision and the period between the procedure and delivery after LEEP, and the results have been inconsistent [8–10].

This study aimed to assess the oncological and obstetric outcomes in patients with CIN treated with LEEP and to investigate the long-term risk factors for recurrence and preterm birth. Additionally, the prognostic value of margin status, when added to HPV positivity for recurrence following LEEP, was also evaluated.

## Methods

### Study protocol and selection criteria

The present study was designed as a single-center, observational, retrospective cohort study. The study was reported following the Strengthening the Reporting of Observational Studies in Epidemiology (STROBE) guidelines and checklist.

We retrospectively identified patients who underwent LEEP for presumed CIN based on cervical biopsy or cytology between January 2011 and December 2019 at our hospital. The inclusion criteria included all patients with CIN who underwent LEEP during the study period. Patients were excluded if the final diagnosis was invasive cervical cancer or adenocarcinoma in situ, if data on human papillomavirus (HPV) status or cervical cytology following LEEP were missing, or if there was another coexisting malignant disease. In cases where the pre- and post-operative biopsy results differed, the worse biopsy result was selected as the final diagnosis.

In reviewing the patients’ medical records, data regarding age, menopausal status, parity, medical history, pre-and post-treatment HPV, cervical cytology, and histopathological results of LEEP, including surgical margin and recurrence status, were collected. For patients who subsequently delivered to our hospital following LEEP, information on pregnancy complications, outcomes, and a history of preterm birth were also obtained.

### Ethics approval and consent to participate

This study was approved by the Institutional Review Board (IRB) of Jeju National University Hospital (IRB number:2022-11-004) and was performed in accordance with the Declaration of Helsinki. The requirement for informed written consent was waived by IRB of Jeju National University Hospital because this retrospective study used existing clinical data and medical records.

### Study outcomes

All patients were assessed using HPV testing using real time PCR (PANA RealTyper HPV kit) and/or cervical cytology within 6–12 months of the LEEP. Vaginal cytology was performed instead of cervical cytology in patients who underwent hysterectomy during the follow-up period. Patients with abnormal cytology or positive HPV results underwent colposcopy and biopsy, if needed. Recurrence was defined as low-grade squamous intraepithelial lesion (LSIL) or worse on cervical or vaginal biopsy or cytology after LEEP. Residual disease was defined as residual LSIL or worse lesions of the cervix in patients who underwent LEEP or hysterectomy within 3 months of LEEP.

### Statistical analysis

Descriptive statistics were used to summarize patient characteristics. Categorical variables were expressed as counts and percentages, and continuous variables were described as mean ± standard deviation or median (interquartile range). Pearson’s Chi-square or Fisher’s exact test was used for categorical variables. Student’s t-test or Wilcoxon Rank-Sum test was used for continuous variables. We calculated the hazard ratio (HR) and 95% confidence interval (CI) for recurrent or residual disease using the Cox proportional hazards model with backward elimination. Kaplan-Meier curves were generated to assess disease-free survival (DFS) to surgical margin status (positive vs. negative) and HPV positivity. DFS was compared using the log-rank test. We evaluated the accuracy of positive margins and HPV positivity after LEEP by calculating the sensitivity, specificity, positive and negative likelihood ratios, and area under the curve (AUC) of recurrent disease. Statistical value of *P* < 0.05 was considered significant for all tests. Statistical analyses were performed using SAS (version 9.4; SAS Institute, Cary, NC, USA) and R (version 3.6.1).

## Results

### Study population

A total of 385 patients who underwent the LEEP between January 2011 and December 2019 were analyzed. All patients were treated using the top-hat method, with endocervical tissue excision immediately following exocervical excision. Forty-six (11.9%) patients had positive surgical margins on LEEP pathology. The median follow-up time for patients with positive margins was longer than that of patients with negative margins; 44.68(IQR 33.30, 56.73) months and 32.87(IQR 12.17, 58.83) months, respectively), although this difference was not statistically significant. There were no significant differences in age, menopausal status, parity, HPV or cervical cytology, and final diagnosis between the two groups regarding surgical margins (Table 1). Among the 46 patients with positive margins, 24 (52.2%) underwent routine surveillance, 19 (41.3%) underwent LEEP again, and three (6.5%) underwent hysterectomy within 3 months after LEEP at the discretion of the physician. Seven (31.8%) cases of residual disease were observed, all of which were HSIL in the specimens after LEEP or hysterectomy.

**Table 1:** Patient characteristics

	Total (*n* = 385)	Margin (−) (*n* = 339)	Margin (+) (*n* = 46)	*P*-value
Age, years, mean ± SD	43.81 ± 11.88	43.45 ± 11.78	46.41 ± 12.42	0.113
Age ≤ 40	154 (40)	138 (40.71)	16 (34.78)	0.441
Age > 40	231 (60)	201 (59.29)	30 (65.22)	
Menopause				
No	292 (75.84)	260 (76.7)	32 (69.57)	0.289
Yes	93 (24.16)	79 (23.3)	14 (30.43)	
Parity				
0	74 (19.22)	68 (20.06)	6 (13.04)	0.508
1	75 (19.48)	66 (19.47)	9 (19.57)	
≥ 2	236 (61.3)	205 (60.47)	31 (67.39)	
HPV status before LEEP				
Negative	26 (6.75)	26 (7.67)	0 (0)	0.071
Positive	319 (82.86)	280 (82.6)	39 (84.78)	
Unknown	40 (10.39)	33 (9.73)	7 (15.22)	
Cervical cytology before LEEP				
Normal, RCC	6 (1.56)	6 (1.77)	0 (0)	0.072
ASCUS	83 (21.56)	76 (22.42)	7 (15.22)	
LSIL	77 (20)	72 (21.24)	5 (10.87)	
HSIL	155 (40.26)	134 (39.53)	21 (45.65)	
Etc	64 (16.62)	51 (15.04)	13 (28.26)	
Final diagnosis (worst)				
Chronic cervicitis	19 (4.94)	18 (5.31)	1 (2.17)	0.516
LSIL	20 (5.19)	19 (5.6)	1 (2.17)	
HSIL	346 (89.87)	302 (89.09)	44 (95.65)	
HPV status after LEEP				
Negative	267 (69.35)	236 (69.62)	31 (67.39)	0.759
Positive	118 (30.65)	103 (30.38)	15 (32.61)	
Cervical cytology after LEEP (worst)				
Normal, RCC	279 (72.47)	253 (74.63)	26 (56.52)	0.070
ASCUS	51 (13.25)	41 (12.09)	10 (21.74)	
LSIL	34 (8.83)	28 (8.26)	6 (13.04)	
HSIL	8 (2.08)	7 (2.06)	1 (2.17)	
Etc	13 (3.38)	10 (2.95)	3 (6.52)	
f/u time (month), median(IQR)	34.57 (12.20, 58.40)	32.87 (12.17, 58.83)	44.68 (33.30, 56.73)	0.078

### Oncologic outcomes

Fifty-two (13.5%) patients experienced recurrent or residual CIN1 + disease and 34 (8.8%) experienced CIN2 + disease after LEEP. In the univariate analysis, menopause, positive surgical margins, and positive HPV status following LEEP were significantly associated with CIN1 + recurrence (Table 2). Multivariate analysis revealed that positive surgical margins and postoperative HPV detection were independent risk factors for CIN1 + recurrence or residual disease (hazard ratio [HR], 1.948; 95%CI 1.020–3.720], *p*-value 0.043 and HR, 6.848; 95%CI 3.652–12.840], *p*-value < 0.001, respectively).

**Table 2:** Predictors of CIN1 + recurrent or residual disease

	Total	Event (%)	Univariate HR		Multivariate analysis	
			HR (95% CI)	*P*-value	HR (95% CI)	*P*-value
Age						
≤ 40	154	16 (10.39)	1			
> 40	231	36 (15.58)	1.570 (0.870–2.835)	0.134		
Menopause						
No	292	33 (11.3)	1			
Yes	93	19 (20.43)	1.992 (1.129–3.515)	0.017		
Parity						
0	74	8 (10.81)	1	0.069		
1	75	4 (5.33)	0.463 (0.139–1.536)	0.208		
≥ 2	236	40 (16.95)	1.468 (0.686–3.144)	0.323		
Final Diagnosis						
Chronic cervicitis	19	3 (15.79)	1	0.973		
LSIL	20	2 (10)	0.843 (0.140–5.069)	0.852		
HSIL	346	47 (13.58)	0.998 (0.310–3.218)	0.998		
Margin						
Negative	339	40 (11.8)	1		1	
Positive	46	12 (26.09)	1.952 (1.020–3.735)	0.043	1.948 (1.020–3.720)	0.043
Exo (+)	9	3 (33.33)	2.782 (0.859–9.008)	0.088		
Endo (+)	32	9 (28.13)	2.061 (0.993–4.277)	0.052		
Both (+)	5	0 (0)	0.000 (0.000–0.000)	0.987		
HPV status after LEEP						
Negative	267	13 (4.87)	1		1	
Positive	118	39 (33.05)	6.843 (3.650-12.827)	0.000	6.848 (3.652–12.840)	0.000

**Fig. 1:**
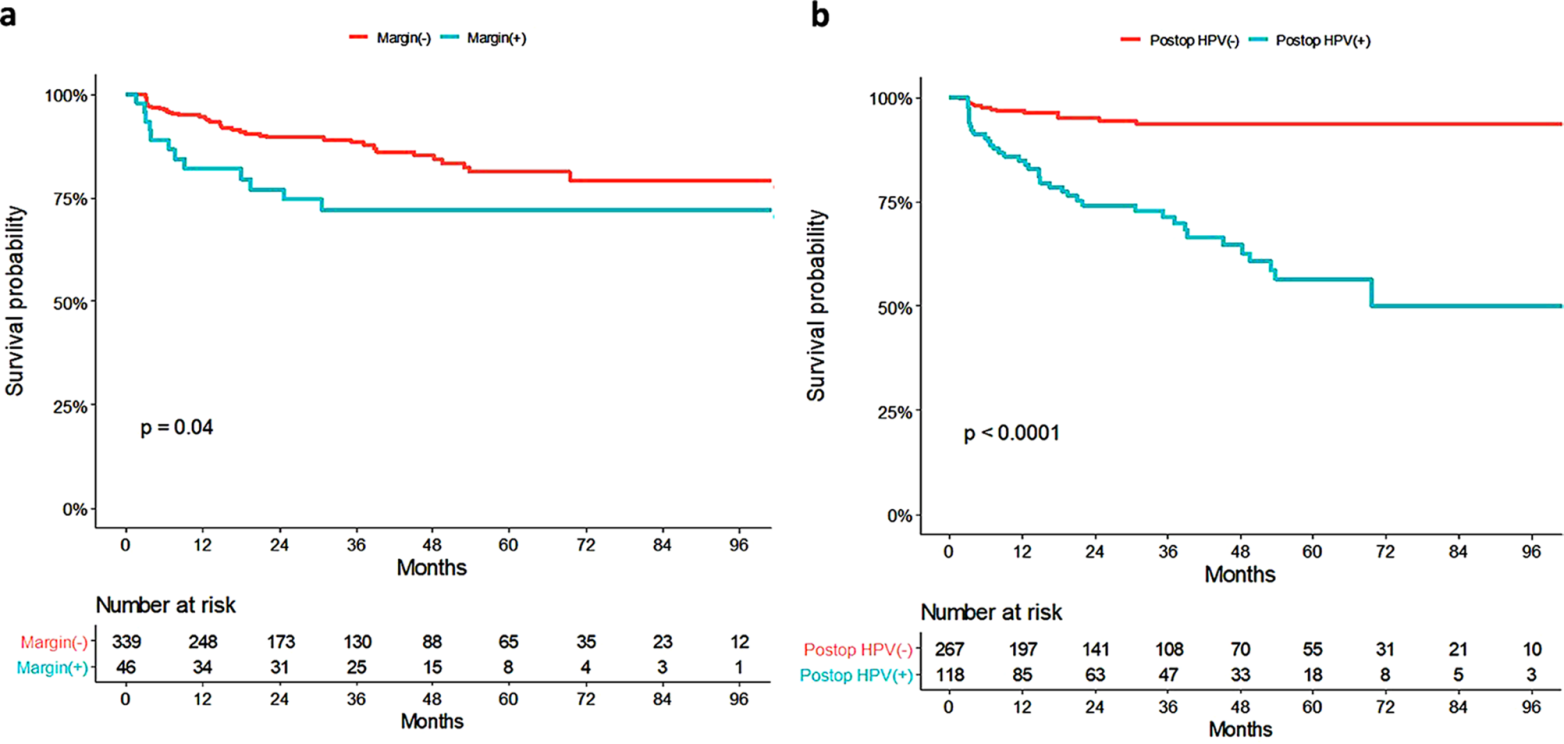
Disease free survival according to (**A**) margin status and (**B**) HPV positivity after LEEP

Figure 1 shows the DFS according to margin status and HPV positivity after LEEP. Positive surgical margins and HPV detection after LEEP were identified as poor prognostic factors for DFS in the log-rank tests (*p* = 0.04 and *p* < 0.0001, respectively).

Table 3 represents the sensitivity, specificity, PPV, NPV, and likelihood ratio of margins or HPV infection as predictors of CIN1 + recurrence or residual disease. The specificity of positive margin and HPV detection after LEEP was 89.79 (95%CI 86.07–92.6) and 76.28 (95% CI 71.42–80.53), respectively. The addition of HPV status to margin status improves specificity as 97.3 (95% CI 94.94–98.57); however, AUC was not improved.

**Table 3:** Sensitivity, specificity, PPV, NPV, likelihood, and AUC

	Sensitivity		Specificity		PPV		NPV		PLR	NLR	AUC	95% CI
Margin status												
Margin(+)	12/52	23.08 (13.72–36.13)	299/333	89.79 (86.07–92.6)	12/46	26.09 (15.6–40.26)	299/339	88.2 (84.33–91.21)	2.26 (1.253–4.076)	0.857 (0.735–0.999)	0.5643	(0.5043–0.6244)
Exo(+), Endo(-)	3/52	5.77 (1.98–15.64)	327/333	98.2 (96.13–99.17)	3/9	33.33 (12.06–64.58)	327/376	86.97 (83.19–90)	3.202 (0.826–12.41)	0.96 (0.896–1.028)		
Exo(-), Endo(+)	9/52	17.31 (9.38–29.73)	310/333	93.09 (89.85–95.35)	9/32	28.13 (15.56–45.37)	310/353	87.82 (83.99–90.83)	2.506 (1.228–5.112)	0.888 (0.782–1.009)		
Both (+)	0/52	0 (0–6.88)	328/333	98.5 (96.53–99.36)	0/5	0 (0–43.45)	328/380	86.32 (82.49–89.41)		1.015 (1.002–1.029)		
HPV status (postop)												
HPV+	39/52	75 (61.79–84.77)	254/333	76.28 (71.42–80.53)	39/118	33.05 (25.22–41.95)	254/267	95.13 (91.85–97.13)	3.161 (2.466–4.053)	0.328 (0.204–0.527)	0.7564	(0.697–0.8201)
HPV (+) and margin(+)	6/52	11.54 (5.4–22.97)	324/333	97.3 (94.94–98.57)	6/15	40 (19.82–64.25)	324/370	87.57 (83.81–90.55)	4.269 (1.585–11.499)	0.909 (0.823–1.005)	0.5442	(0.4995–0.5889)
HPV (+) and margin (-)	33/52	63.46 (49.87–75.2)	263/333	78.98 (74.28–83.01)	33/103	32.04 (23.81–41.56)	263/282	93.26 (89.72–95.64)	3.019 (2.252–4.047)	0.463 (0.322–0.665)		
HPV (-) and margin (+)	6/52	11.54 (5.4–22.97)	308/333	92.49 (89.15–94.86)	6/31	19.35 (9.19–36.28)	308/354	87.01 (83.1–90.11)	1.537 (0.662–3.566)	0.956 (0.863–1.06)		
HPV (-) and margin (-)	7/52	13.46 (6.68–25.27)	104/333	31.23 (26.49–36.4)	7/236	2.97 (1.44–5.99)	104/149	69.8 (62.01–76.6)	0.196 (0.098–0.391)	2.771 (2.287–3.358)		

### Obstetric outcomes

A total of 31 patients subsequently delivered at our hospital after LEEP. Among them, 14 (45.1%) experienced preterm-related complications, such as a short cervix, incompetent internal os of cervix (IIOC), preterm labor, and preterm premature membrane rupture. Eight patients (25.8%) had premature birth before the age of 37 weeks. The median gestational age was 38.1 weeks (range 34.1–40.6 weeks). The median duration between the LEEP and delivery was 36.6 months (range 13.6–87.3 months). A history of preterm birth and duration between LEEP and delivery were significantly associated with preterm-related complications. However, the duration between the LEEP and delivery was not significantly related to preterm birth before 37 weeks (Table 4).

**Table 4:** Risk factors for preterm complications and preterm delivery

	Total (*n* = 31)	Preterm related complications			preterm birth		
		no (*n* = 17)	yes (*n* = 14)	*P* value	no (*n* = 23)	yes (*n* = 8)	*P* value
age at delivery	33.42 ± 3.94	33.04 ± 3.71	33.87 ± 4.29	0.567	33.88 ± 3.94	32.08 ± 3.86	0.274
parity at delivery							
0	10 (32.26)	6 (35.29)	4 (28.57)	0.660	7 (30.43)	3 (37.5)	0.269
1	10 (32.26)	4 (23.53)	6 (42.86)		6 (26.09)	4 (50)	
>=2	11 (35.48)	7 (41.18)	4 (28.57)		10 (43.48)	1 (12.5)	
Previous preterm birth history							
0	27 (87.1)	17 (100)	10 (71.43)	0.032	22 (95.65)	5 (62.5)	0.043
1	4 (12.9)	0 (0)	4 (28.57)		1 (4.35)	3 (37.5)	
The duration between LEEP and delivery (day)	1149.94 ± 713.74	784.82 ± 314.76	1593.29 ± 817.09	0.009	1013.57 ± 585.25	1542 ± 932.71	0.214
preop HPV							
Negative	27 (87.1)	13 (76.47)	14 (100)	0.108	19 (82.61)	8 (100)	0.550
Positive	4 (12.9)	4 (23.53)	0 (0)		4 (17.39)	0 (0)	
Final Diagnosis							
Negative(cervicitis)	2 (6.45)	2 (11.76)	0 (0)	0.239	2 (8.7)	0 (0)	1.000
LSIL	2 (6.45)	2 (11.76)	0 (0)		2 (8.7)	0 (0)	
HSIL	27 (87.1)	13 (76.47)	14 (100)		19 (82.61)	8 (100)	
Margin							
Negative	27 (87.1)	14 (82.35)	13 (92.86)	0.607	19 (82.61)	8 (100)	0.550
Positive	4 (12.9)	3 (17.65)	1 (7.14)		4 (17.39)	0 (0)	
Exo (+)	2 (50)	2 (66.67)	0 (0)		2 (50)		
Endo (+)	1 (25)	0 (0)	1 (100)		1 (25)		
Both (+)	1 (25)	1 (33.33)	0 (0)		1 (25)		
Postop HPV							
Negative	22 (70.97)	12 (70.59)	10 (71.43)	1.000	15 (65.22)	7 (87.5)	0.379
Positive	9 (29.03)	5 (29.41)	4 (28.57)		8 (34.78)	1 (12.5)	
excised lesion size							
Depth	1.04 ± 0.37	1.04 ± 0.4	1.04 ± 0.35	0.808	1.06 ± 0.37	0.98 ± 0.4	0.800
Maximal length	2.63 ± 0.51	2.56 ± 0.43	2.71 ± 0.6	0.409	2.63 ± 0.48	2.62 ± 0.63	0.821

## Discussion

Our results indicate that surgical margin involvement during the LEEP and HPV positivity after the LEEP are independent predictors of treatment failure in patients with CIN. In addition, 32% of the patients with positive resection margins who underwent re-excision were found to have residual disease. HPV status following LEEP was a stronger predictor of treatment failure than margin status, and combining the two factors did not improve the prediction accuracy.

Most previous studies included CIN2 + disease in recurrence [1, 2, 11–13], whereas our study included CIN1 in recurrence. We acknowledge CIN2 + recurrence is more critical than CIN1 recurrence due to the necessity for immediate treatment. In the literature, although most CIN 2 + recurrences are diagnosed within 2 years of treatment, CIN 1 disease should also be followed up with caution among patients who undergo LEEP [14]. 35% of our recurrences were diagnosed as CIN 1 disease. CIN 1 does not require immediate intervention; nevertheless, its significance lies in the need for careful surveillance, the associated medical costs, and the resulting patient concern. Therefore, we defined CIN1 + disease as recurrence in this study, even though patients with CIN1 had a low rate of progression to CIN2 + disease [15, 16].

Positive high-risk HPV status and surgical margin involvement are well-established risk factors for recurrence after LEEP [1, 2, 11–13, 17–19], consistent with our findings. Notably, persistent infection of high-risk HPV is strongly correlated with recurrence in previous study [20]. While we analyzed both low- and high-risk HPV, HPV infection remained a robust risk factor for recurrence. In earlier studies, the involvement of the endocervical margin was an independent risk factor for recurrence, and the risk did not increase when only the exocervical margins were positive [2]. However, we did not observe a significant increase in risk according to the exocervical/endocervical margin status. We speculate that these results indicate an influence from bias, potentially arising from the inherent inaccuracy in the assessment of surgical margins. Our findings support the prior meta-analysis, underscoring the difficulty of predicting patient recurrence solely based on surgical margins after LEEP due to the low reproducibility and imprecision in the assessment of the resection margins [12].

We found that HPV positivity after LEEP was a more accurate predictive marker for recurrence with an AUC of 0.76 compared with a margin status of 0.56. Our findings are in concordance with the previous meta-analytic study [12]. Adding postoperative HPV status to margin status improved specificity but lowered sensitivity, which is consistent with previous studies [11, 13]. These studies showed that adding margin status to HPV tests did not substantially improve prediction accuracy [11, 13], and our results support these findings, as the AUC of combined HPV and margin status was lower than the AUC of HPV and margin status individually. Based on these findings, we suggest that HPV-based follow-up is important for surveillance after LEEP.

In our study, 22 patients with positive margins who had no desire for fertility underwent LEEP again or hysterectomy within 3 months after LEEP. These decisions deviate from the established guidelines that recommend against retreatment for margin-positive cases [12]; however, they were made based on the physician’s discretion, considering both risk factors and patient concerns. Numerous studies have demonstrated the association between adverse obstetric outcomes and LEEP treatment [19, 21–23]. Therefore, unnecessary retreatment or hysterectomy in margin-positive patients who wish to preserve fertility should be avoided.

The Preterm birth rate in our study was 25.8% among patients who delivered at our hospital. This should be interpreted cautiously because in a total of 385 patients, including 154 who underwent LEEP under the age of 40 years, only 31 delivered at our hospital. These patients were presumed to have high-risk pregnancies considering that our institution is a university hospital. Obstetric outcomes after the LEEP were not investigated in the entire study population. However, given the regional characteristics of the island with low population movement, it can be assumed that most patients with complications related to premature birth were hospitalized at our hospital.

Several studies have reported that the height of the tissue during conization is associated with preterm delivery [24–26]. Our results showed that there was no significant association between the depth of tissue and preterm delivery; however, we infer that the relatively short depth of tissue (mean 1.04 ± 0.37 cm) in our data compared to another study (mean > 1.3 cm) [24, 25] may have influenced the results. A previous study showed that a cone height above 1.7 cm was associated with a greater risk of premature rupture of membranes [24], but there was only one case of tissue height above 1.7 cm in our study.

There is controversy in the literature regarding whether the interval between conization and delivery is a risk factor for subsequent preterm delivery [26–28]. In our study, a shorter interval from LEEP to delivery was associated with preterm-related complications, although it was unrelated to the actual preterm delivery. Considering that the mean interval between LEEP and delivery was relatively longer than that in previous studies [26, 27], the increase in preterm-related complications, even though the risk of preterm delivery did not increase, highlights the long-term adverse obstetric effects of LEEP.

The major limitation of our study is that we collected and analyzed data without distinguishing specific HPV genotypes. Because many previous studies elucidated the significance of same-genotype HPV persistence [15, 16, 29–32], the inability to confirm HPV genotype persistence in our study might have caused a bias. Additionally, we investigated the risk factors for the recurrence of CIN1 + disease but did not analyze CIN2 + recurrence separately because of the small number of events. However, this was a long-term follow-up study that observed oncological and obstetric outcomes due to the regional characteristics of the island. Furthermore, we found that HPV positivity after LEEP was a more accurate predictive marker for recurrence compared to surgical margin. This facilitates postoperative patient counseling and surveillance after LEEP and highlights areas for future research in cervical cancer prevention.

## Conclusions

Our study highlights that the HPV status after LEEP and margin involvement are independent risk factors for recurrence in patients with CIN who undergo LEEP. Surgeons should make every effort to obtain free margins during LEEP, and human papillomavirus testing may be the most useful method for predicting recurrence during surveillance.

## Data Availability

The datasets used and/or analyzed during the current study available from the corresponding author on reasonable request.

## Abbreviations

LEEP: Loop Electrosurgical Excision Procedure
HPV: Human Papilloma Virus
CIN: Cervical Intraepithelial Neoplasia
